# Comparative Genomic Analysis Reveals a Critical Role of *De Novo* Nucleotide Biosynthesis for *Saccharomyces cerevisiae* Virulence

**DOI:** 10.1371/journal.pone.0122382

**Published:** 2015-03-27

**Authors:** Roberto Pérez-Torrado, Silvia Llopis, Benedetta Perrone, Rocío Gómez-Pastor, Bernhard Hube, Amparo Querol

**Affiliations:** 1 Instituto de Agroquímica y Tecnología de los Alimentos, CSIC, Valencia, Spain; 2 Department of Agricultural, Forest and Food Sciences Microbiology and Food Technologies Unit University of Torino, Torino, Italy; 3 Department of Microbial Pathogenicity Mechanisms, Leibniz Institute for Natural Product Research and Infection Biology, Hans-Knoell-Institute (HKI), Jena, Germany; 4 Friedrich Schiller University, Jena, Germany; Worcester Polytechnic Institute, UNITED STATES

## Abstract

In recent years, the number of human infection cases produced by the food related species *Saccharomyces cerevisiae* has increased. Whereas many strains of this species are considered safe, other ‘opportunistic’ strains show a high degree of potential virulence attributes and can cause infections in immunocompromised patients. Here we studied the genetic characteristics of selected opportunistic strains isolated from dietary supplements and also from patients by array comparative genomic hybridization. Our results show increased copy numbers of *IMD* genes in opportunistic strains, which are implicated in the *de novo* biosynthesis of the purine nucleotides pathway. The importance of this pathway for virulence of *S*. *cerevisiae* was confirmed by infections in immunodeficient murine models using a *GUA1* mutant, a key gene of this pathway. We show that exogenous guanine, an end product of this pathway in its triphosphorylated form, increases the survival of yeast strains in *ex vivo* blood infections. Finally, we show the importance of the DNA damage response that activates dNTP biosynthesis in yeast cells during *ex vivo* blood infections. We conclude that opportunistic yeasts may use an enhanced *de novo* biosynthesis of the purine nucleotides pathway to increase survival and favor infections in the host.

## Introduction

The yeast *Saccharomyces cerevisiae* can be found naturally in many niches in the environment, but it is most commonly known for its role as “baker’s yeast” in either the traditional or industrial fermentative production of bread, beer or wine. It can be found in many dietary supplements as the main component and a part of these yeast cells are alive. It has also been used as a nutritional supplement and as an agent to treat antibiotic-related diarrhea, commercialized as *Saccharomyces boulardii* [1]. Classically, *S*. *cerevisiae* has been considered a non-pathogenic and safe organism. However, in the last few years, cases of diagnosed *S*. *cerevisiae* infections have increased, probably due to an increase in the numbers of immunocompromised patients and the progress made in diagnostic methodologies, including microbial identification by molecular techniques. *S*. *cerevisiae* has been identified in a wide variety of infections, ranging from cutaneous infections and vaginitis in healthy individuals, to systemic bloodstream infections and infections of vital organs in immunocompromised and critically ill patients [1–3]. Infected patients are premature children, elderly people or patients suffering from immunosuppression due to AIDS, treatment with immunosuppressive agents, or other conditions associated with an insufficient immune response. Moreover, severe infections with *S*. *cerevisiae* have been occasionally reported in patients with no obvious predisposing factors [4,5]. All these data have changed the status of *S*. *cerevisiae*, which is now considered to be an emerging opportunistic pathogen [6–9]. Several studies have analyzed the potential virulence of this yeast species *in vitro* [10–13], while others have used *in vivo* models [13–17]. These reports suggest that some clinical, but also non-clinical, *S*. *cerevisiae* strains have the potential to cause disease in murine models regardless of the host’s immune status.

To elucidate the genetic determinants that influence the ability of *S*. *cerevisiae* to cause infection, we selected strains that showed high levels of virulence attributes (pseudohyphal and growth at 42°C, phosphatases and proteases secretion, and the ability to infect and kill mice) independently of the isolation origin [13], [16], [18], and we used these strains for a comparative genetics approach. We observed increased copy number (CN) of the genes related to the *de novo* biosynthesis of the purine nucleotides pathway. One of the key genes of this pathway, which encodes an enzyme catalyzing the second pathway step, is *GUA1*. The importance of this pathway for virulence of *S*. *cerevisiae* was confirmed by experimental infections in immunodeficient murine models using a Δ*gua1* mutant of the clinical strain D14, isolated from a dietary product. We also revealed that exogenous guanine, an end product of the purine nucleotides pathway in its triphosphorylated form, increases the survival of yeast strains in *ex vivo* blood infections. Finally, we show the implication of the DNA damage checkpoint that activates dNTP biosynthesis in yeast cells during blood infections. We conclude that opportunistic yeasts may use an enhanced *de novo* biosynthesis of the purine nucleotides pathway to counteract the immune system of the host. An increased efficiency of dNTP biosynthesis may in turn promote DNA repair after DNA damage produced by neutrophil oxidative bursts.

## Materials and Methods

### Yeast strains and media

Several *S*. *cerevisiae* isolates were used in this work: a clinical vaginal isolate (isolate 60), an isolate from the respiratory tract (isolate 102) [10], a brewer’s strain isolated from a commercial nutritional complement product (D14) [13], auxotrophic laboratory strains W303 (*MATa*; *ura3*-52; *trp1*Δ2; *leu2*-3,112; *his3*-11; *ade2*-1; *can1*-100), BY4741 (*MATahis3*Δ0 *leu2*Δ0 *met15*Δ0 *ura3*Δ, Open Biosystems), Δ*dun1* (*MATa his3*Δ0 *leu2*Δ0 *met15*Δ0 *ura3*Δ, Open Biosystems), W1588-4C (*MAT* **a** *ade2-1 can1-100 his3-11*,*15 leu2-3*,*112 trp1-1 ura3-1 RAD5*) (31), rnr1* (*MAT* **a** *ade2-1 can1-100 his3-11*,*15 leu2-3*,*112 trp1-1 ura3-1 RAD5 rnr1-W688G*) [19] and prototrophic strain S288C (*MATα SUC2 gal2 mal mel flo1 flo8*-1 *hap1*). Isolates 102, 60 and D14 were chosen from a yeast collection of potential pathogenic isolates obtained mainly from hospitals, but also from commercial dietary products [16], [18]. These isolates were proposed to have a higher virulence potential since they showed increased pseudohyphal and 42°C growth [18], augmented phosphatases and proteases secretion [18] and were able to survive and to colonize in the brain and kidney of immunocompetent mice after blood infections, thereby causing morbidity and death among their hosts [13], [16]. In addition, strains 60 and D14 were less efficiently phagocytized by macrophages as compared to other strains [13]. *S*. *cerevisiae* strains were cultured in YPD (1.0% yeast extract, 1.0% Bacto-peptone and 2.0% glucose) and in SC medium (5.6% YNB without amino acids, 1.3% complete amino acid drop out (Formedium, UK)). The corresponding media were supplemented with CuSO_4_ (0–2.0 mM) and also mycophenolic acid (MPA) (7 μg/ml) or methyl methanesulfonate (MMS) (0.004%) or guanine (25 μM) based on previously reported methods [20–22].

The mutant D14Δ*gua1* was obtained by a PCR-based gene-replacement strategy using the subsequent deletion cassettes containing geneticin or hygromycin resistance [23]. The primers used had 40 bp homologous to the gene coding sequence at their 5’ or 3’ end. The amplified PCR products were used for gene disruption, performed by homologous recombination following yeast transformation using the lithium acetate procedure. The PCR reactions were as follows: 2 min at 94°C; 30 cycles of 15 s at 94°C, 30 s at 50°C, 2 min at 72°C and 5 min at 72°C. Cells were transformed with the PCR product following the LiAc protocol (http://home.cc.umanitoba.ca/~gietz/website). After heat shock, cells were incubated for 3 h in YPD liquid medium at 30°C. Finally, transformed cells were selected on YPD plates with geneticin (200 mg/L) or hygromycin. *GUA1* gene deletion was confirmed by a PCR analysis and guanine auxotrophy. Strain D14GUA1 was obtained by transforming strain D14Δgua1 with a plasmid containing a wild-type copy of *GUA1* (pGUA1) and by selecting in YPD with 200 μg/mL of G418 (Sigma). The plasmid obtained from Iglesias-Gato *et al*. [21] was marker-swapped for KANMX by homologous recombination using a cassette obtained by PCR and a pUG6 plasmid as a template.

Yeast growth in the presence of copper salt (0, 0.5, 1 and 2 mM of CuSO_4_) was quantified in microtiter plates on a reader model POLARstar Optima (BGM Labtech, Offenburg, Germany). Wells were filled with the appropriate number of yeast cells and 0.5 ml of YPD medium (with or without copper salt) to reach an initial OD of approximately 0.2 (corresponding to a starting cell number of ∼10^6^ cells/ml). Non-inoculated wells were included for each experimental series to determine, and consequently subtract, noise signals. Growth was monitored by optical density (OD) changes at a wavelength of 600 nm. Measurements were taken every 30 min for 72 h at 28°C (until yeast cells reached the stationary phase) after a 20-second pre-shaking. All experiments were carried out in triplicate and maximal OD was obtained for each condition.

### DNA labeling and competitive genome hybridization

DNA was extracted from yeast strains grown from single colonies in YPD medium following the procedure described by Belloch *et al* [23]. Before the labeling reaction, 4 μg of DNA were treated with RNAse A (1 mg/mL, Roche Diagnostics) for 1 h at 37°C and with Proteinase K (1 mg/ml, Quiagen) for 1.5 h at 37°C with agitation. Phenol extracted DNA was digested with MseI or RsaI (New England Biolabs, Inc.), according to the manufacturer's instructions, and digestions were mixed. The fragmented sample was purified using the QIA-quick gel extraction kit (Qiagen, Germany), heat denatured for 5 min at 100°C, and then cooled on ice. DNA was labeled in 21-μl reaction mixtures by random priming using the BioPrime Array CGH Genomic Labeling System (Invitrogen). Unincorporated nucleotides are removed by filtration through a Qiaquick PCR Purification column (Qiagen). Equal amounts of labeled DNA (1μ) were used as probes to hybridize to the PCR-amplified open reading frames (ORFs) of homoploid *S*. *cerevisiae* S288C DNA spotted onto microarrays (Eurogentec S.A., Belgium). Microarrays were pre-hybridized with 5 ml of pre-hybridization solution (3× SSC (1× SSC is 0.15 M NaCl plus 0.015 M sodium citrate), 0.1% SDS, and 0.1 mg/ml BSA) at 42°C for 1 h. After washing slides with isopropanol and water, samples were centrifuged at 1300 rpm for 10 min at room temp. Then 10 μl of samples and 50 μl of hybridization solution (50% (v/v) formamide, 5x SSC, 0.1% SDS and 0.1 mg/ml DNA from salmon sperm) were denatured (95°C, 1 min) and added to the slides that were incubated for 16 h at 42°C in a Gene Machines Hyb Chamber (Genomic Solutions). Slides were recovered and washed 5 min at 42°C in 1 (2x SSC, 0.1% SDS), 2x10 min in solution 2 (0.1 x SSC, 0.1% SDS), 5x1min in solution 3 (0.1 x SSC) and 5 s in solution 4 (0.01 x SSC). Slides were centrifuged for 10 min at 1300 rpm.

### Microarray scanning and data normalization

Images were acquired using GenePix 4100A (Axon Instruments, Molecular Devices Corp., USA) with a 10-μm resolution using the GenePix Pro 6.1 software (Axon Instruments, Molecular Devices Corp., USA). Raw data were processed using Acuity 4.0 (Axon Instruments, Molecular Devices Corp., USA). All the slide data were normalized with a log_2_(ratio) average and filtered (signal of each channel > 350 and signal/noise ratio > 2.5). Poor or inconsistent signals were not considered for further analysis. Hybridization signals were depicted as the log2 hybridization signal ratio. Genomic material obtained from three biological independent replicates was used to perform hybridizations two or three times, including a dye-swap, and the genes with signal intensities ± 0.5 were considered for further analysis. FDR<0.05 was used to select significant data. Heat map was generated in MeV 4.9 software using developers instructions were each row represents a specific gene and each column represents each strain. The lightest green boxes are the most underrepresented genes and the brightest red being the most overrepresented genes.

### Flow cytometry

Yeasts strains were grown in 10 ml of YPD liquid medium under agitation. Early-stationary-phase cells (10^6^ cells ml^−1^) were harvested by centrifugation, washed and fixed in 70% ethanol at 4°C for 5 min. Fixed cells were harvested by centrifugation and resuspended in 0.01 M phosphate-buffered saline buffer (pH 7.2) containing 400 μl of RNase (10 mg ml^−1^). After incubation at 37°C for 30 min, cells were harvested by centrifugation and re-suspended in 950 μl of 0.01 M phosphate-buffered saline buffer (pH 7.2) containing 50 μl of propidium iodide (0.005%). Samples were analyzed using a flow cytometer FACScan analyzer (Becton Dickinson). DNA content values were scored on the basis of the fluorescence intensity of the first peak by comparing with the haploid W303 and diploid S288c strains.

### qRT-PCR

To validate the microarrays results, the DNA from the five strains was isolated and the copy number of the three genes was quantified. The genes selected were *IMD2*, *IMD3* and *SAM3* with criteria explained below. qRT-PCR was performed with gene-specific primers (200 nM) (IMD2-F: AACATTCCTGTCAAGACATCGG, IMD2-R: TCAGTTATGTAAACGCTTTTCGTAA, IMD3-F: CGGTTTACAACATTCTTGTCAAGAC, IMD3-R: AAACGCTTTTCGTAAGAATGTAAGT, SAM3-F: GCGGGTGAAATATACGTATCGG, SAM3-R: ATTCTCCACGGAACACCCAGTA, ACT1F: TACAACTCCATCATGAAGTG and ACT1-R: GCCAAAGCGGTGATTTCC) in a 20 μl reaction using Light Cycler Fast Start DNA Master PLUS SYBR green (Roche Applied Science, Germany) in a LightCycler 2.0 System (Roche Applied Science, Germany) device. All the samples were processed for the melting curve analysis, amplification efficiency and DNA concentration determination using the LightCycler 2.0 System. A mix of all the samples and serial dilutions (10^-1^ to 10^-5^) was utilized as the standard curve. Data were normalized with the *ACT1* gene to correct for the DNA content in each sample. Data are presented as the average ± standard deviations of three biological replicates.

### Experimental infection

*S*. *cerevisiae* strains were grown at 30°C on YPD. After 24 h, cells were harvested, washed twice with sterile phosphate-buffered saline (PBS) and diluted to the desired density. Healthy mice were infected by intravenous inoculation into the tail vein with 2 × 10^7^ viable CFU of yeast in a 0.2-ml volume of PBS, as previously described [15]. Twenty-four DBA/2 mice, aged 7–8 weeks, were respectively used (8 mice per *S*. *cerevisiae* strain). Mice were housed in groups of five in individually ventilated cages and cared for in strict accordance with the principles outlined in the *European Convention for the Protection of Vertebrate Animals Used for Experimental and Other Scientific Purposes* (http://conventions.coe.int/Treaty/en/Treaties/Html/123.htm). Experiments were approved by the governmental ethics committee (Comité Ético de Experimentación Animal, Universitat de Valencia). Neutropenia in mice was produced by an intraperitoneal injection of 150 mg/kg of cyclophosphamide (Sigma-Aldrich) 1 day before inoculation with each *S*. *cerevisiae* strain. Five days later, all the animals received a second dose of cyclophosphamide (150 mg/kg) to maintain neutropenia until the end of the assay. Similar treatments have demonstrated that severe neutropenia can be achieved and maintained [24]. The infected animals were visually monitored at least twice daily and they were humanely euthanized by cervical dislocation when any apparent signs of suffering were found. Mouse survival was analyzed by using Kaplan-Meyer curves and the log-rank test. No adverse events occurred during the assay.

### Survival assay in human blood

To investigate the survival of yeast strains in blood we used the method previously described [25]. Yeast strains were grown overnight in YPD medium at 30°C. Cells were washed once and suspended in PBS buffer (phosphate-buffered saline: 150 mM NaCl, 16 mM Na_2_HPO_4_, 4 mM NaH_2_PO_4_, pH 7.4) at a density of 5 x 10^7^ cells/ml. Human peripheral venous blood was collected from healthy volunteers by venipuncture using ammonium heparin syringes (Monovette, Sarstedt) as described [25]. Blood was obtained from healthy human donors with written informed consent. The blood donation protocol and use of blood for this study were approved by the institutional ethics committee (Comité Ético del Insituto de Agroquímica y Técnología de los Alimentos, CSIC). After leukocyte count with Neubauer chamber, yeast cells were inoculated (1:1 ratio of yeast:leukocytes) in blood and incubated for 90 min at 37°C as described [25]. Then, 100 μl of the 10^-3^ to 10^-4^dilutions were spread on YPD plates and incubated for 48 h at 30°C. Colony-forming units (cfu) were counted and relative percentages of survival were determined as follows: (((cfu/cfu_t = 0_))/((cfu/cfu_t = 0_)_control_)*100)-100. To avoid effects of individual blood differences, survival was normalized to the reference strain included in each set of experiments. Each strain was tested 3 times. Data were represented as averages ± standard deviations.

## Results

Virulent strains of *S*. *cerevisiae* must have specific attributes which contribute to their potential to cause opportunistic infections. To search for specific features of virulent strains of *S*. *cerevisiae*, we studied their genome structures by microarray CGH. This technique allowed us to identify those genes with copy number variations (CNV) in the opportunistic strains compared with non-virulent strains. We selected three different opportunistic *S*. *cerevisiae* strains (D14, 60, 102) which have been shown to exhibit high levels of virulence *in vitro* and *in vivo*, presented enhanced survival rates in blood, increased burdens in brain and kidney, and were able to kill mice [13], [16], [18]. Since the ploidy of the different strains is important for interpreting CGH data, we analyzed ploidy by flow cytometry. The results suggested that strains 102 (2.00 ± 0.07) and D14 (2.16 ± 0.07) are diploids (considering 20% deviation plausible due to experimental error), whereas the result of strain 60 (2.41 ± 0.05) indicate that it is a diploid strain with additional copies of some chromosomes. The labeled DNA from the four strains was hybridized with DNA from control strain S288C to a standard cDNA microarray containing cDNA of the S288C genome. The signals obtained were filtered and normalized. The whole dataset is deposited in GEO Database (accession number GSE53440) using standard MIAME criteria.

### Comparative genomic hybridization of opportunistic strains

**Fig 1:**
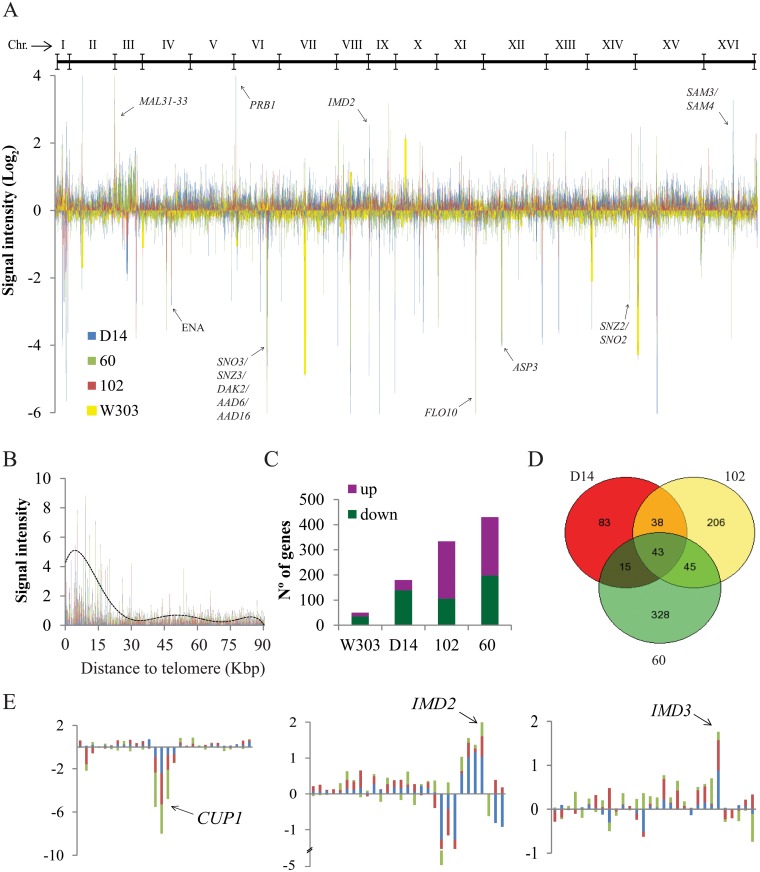
Comparative genomic hybrization of opportunistic strains. D14, 60 and 102 were compared to laboratory strain W303 hybridizing DNA to S288C cDNA microarray. A) Log2 of normalized and filtered data of the four strains is represented according its chromosomal disposition in stack bar graph. Specific genes are depicted with an arrow. B) Absolute signal intensity of genes is represented against distance to telomere. Discontinuous line represents sliding window average (10x) of values in 1 Kbp. C) Number of genes in increased or decreased CNV represented for each strain. D) Venn diagram showing the number of specific or common genes for strains D14, 103 and 60. E) Local zoom of general graph presented in A) for genes *IMD3*, *IMD2* and *CUP1* chromosomal regions for opportunistic strains.

When we analyzed the CGH data in a genome-wide graph with all the strains, including control strain W303 (Fig. 1A), we observed a significant amount of CNV in the subtelomeric regions (22.8%). When we analyzed the absolute signal intensity of the genes in relation to the distances to telomeres (Fig. 1B), we observed that CNV significantly accumulated in the first 30 kb, which matches the subtelomeric region. The sliding window analysis, which averaged signal intensity (1 Kb), also revealed a significant increase in the subtelomeric region. These observations are consistent with previous CGH studies which compared the genome of *S*. *cerevisiae* strains of different origins [26], [27]. As expected, W303, whose genome is 85% isogenic to S288C [28], was the strain that showed less CN (50) as compared to the opportunistic strains D14 (180), 102 (334) and 60 (430),with differences found mainly in the number of increased CN (Fig. 1C). To confirm these data, we tested for the presence of W303 in the sequenced genome (www.yeastgenome.org) of the first 25 genes with less relative intensity data, and most of them [28] were absent. These results also confirmed that some genes, previously described as highly variable among strains of different sources [26], [27], for example, members of the *PAU*, *MAL*, *AAD* and *FLO* gene families, the *ASP3* and *ENA* clusters, the *SNO/SNZ* and *SOR/MPH* genes and other genes, i.e., *DAK2* or *AGP3*, showed altered CN in opportunistic strains. Among these genes, whose numbers had increased in opportunistic strains, we found several genes related to DNA damage repair, such as *DDR48*, *CAC2* and *RRD1* in strain 102 and *RAD18*, *POL4*, *MSH2* and *IMP2* in strain 60. One interesting result was the strong increase in the CNV of *PRB1*, which encodes a protease whose protein abundance increased after DNA damage in strains 60 and 102. Another observation was the increase in the CNV for gene *YAR068W*. An overexpression of this uncharacterized gene has been seen causing enhanced resistance to antifungal drugs, such as ketoconazole, miconazole and benomyl [29].

**Fig 2:**
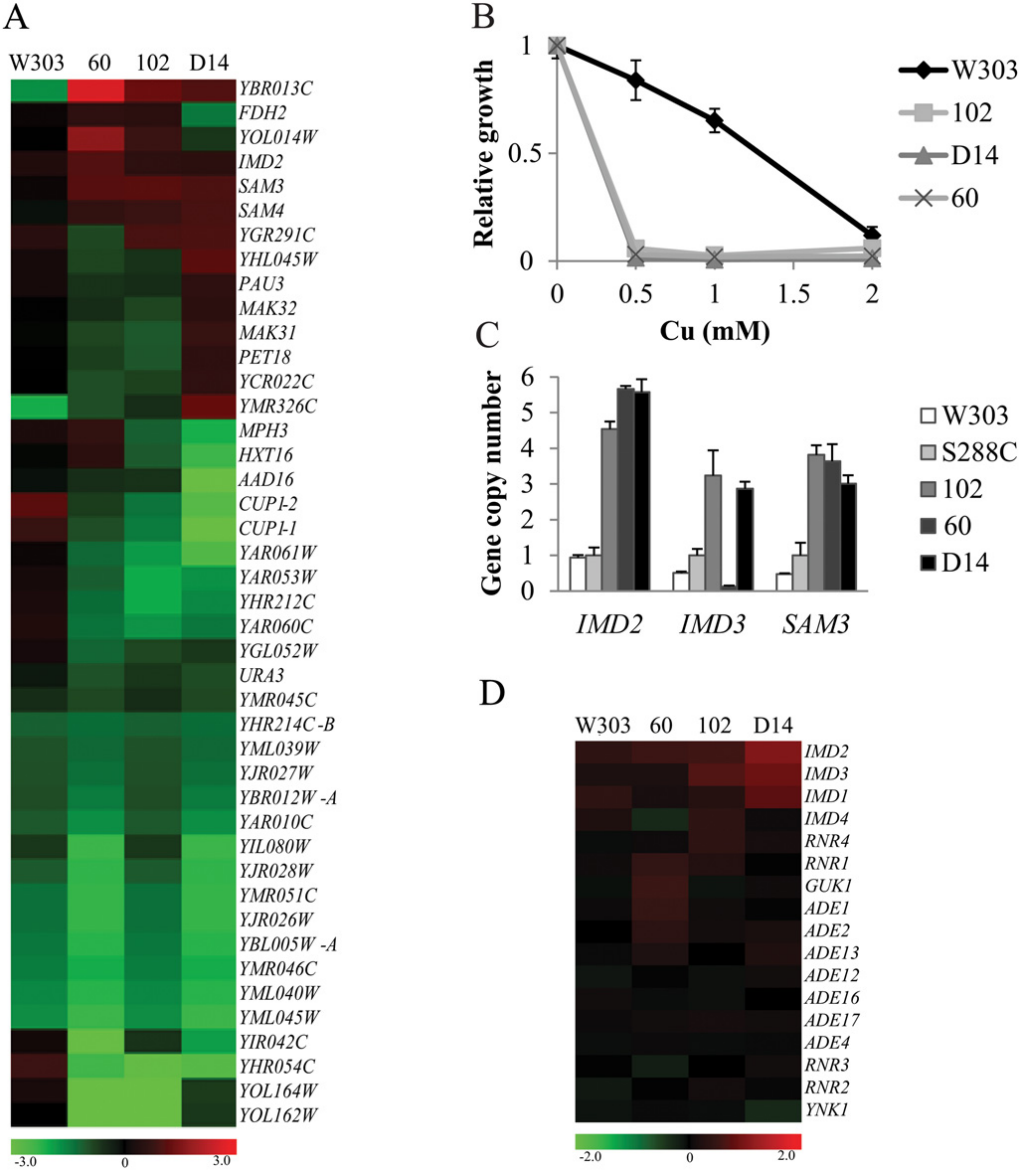
Opportunistic strains showed altered CN of specific genes. A) Genes that showed altered CNV in the three opportunistic strains were clustered and the heat map obtained (Mev software) is represented. B) Evaluation of growth with different concentrations of copper was performed in microtiter plates in YPD media. Maximal growth for each Cu concentration was relativized to YPD without Cu for each strain. Average and standard deviation of triplicates is represented C) Gene copy number was determined by qPCR. Data was normalized with haploid strain S288C. Average and standard deviation of triplicates is represented. D) Heat map detail of genes that belong to de novo purine biosynthetic pathway (Mev software).

We searched for genes with CNV in all investigated strains and found that 45 genes were common for the opportunistic yeasts (Fig. 1D). A hierarchical clustering of this core set of CNV-enriched genes (Fig. 2A) revealed a clear difference between opportunistic and laboratory strains. All except 14 transposon genes and two other genes, the genes showed opposite or different CNV values in the opportunistic strains as compared to W303. Twenty-seven of these 45 genes presented decreased CN, and four increased and 14 variable values among the different strains. Interestingly, 31.1% of the core genes were transposons, a fact that reflects one of the most important differences observed when comparing genomes of different strains. Next, we focused on those genes showing an altered CN in the opportunistic strains when compared to laboratory strains. Among this group of genes, we focused our study on the *CUP1* cluster, which displayed a decreased CN in the opportunistic strains when compared with laboratory strains. The SAM cluster and *IMD2* had an increased CN in the opportunistic strains *vs*. laboratory strains.

### Genetic variability in the *CUP1* cluster

The *CUP1* cluster contains a duplication of the *CUP1* gene flanking an uncharacterized ORF (*CUP1-1*, *YHR054C* and *CUP1-2*). This cluster shows a decreased CN in the opportunistic strains when compared with laboratory strains (Fig. 1E). High variability in CN has been described for this cluster, which is probably the result of recombination events between duplicates [27]. Since the gene copy number correlates with Cu resistance [30], we studied the growth of both, the opportunistic strains and W303, in minimal media with a Cu concentration range in order to validate the CGH analysis with phenotypical data. The results obtained (Fig. 2B) confirm the CGH data as none of the opportunistic strains was able to grow, not even at the lowest Cu concentration. Conversely, lab strain W303, which has a larger number of *CUP1* copies, was able to grow with 1 mM of Cu.

### Alteration in S-adenosylmethionine metabolic genes

Two of the genes with an increased CN in the opportunistic *S*. *cerevisiae* strains were *SAM3* and *SAM4*, which encode a high-affinity S-adenosylmethionine (SAM) permease and an S-adenosylmethionine-homocysteine methyltransferase, respectively. We studied the genomic content of *SAM3* in the different strains with qPCR in more detail (Fig. 2C). The results confirm the CGH data and suggest at least one more copy of the gene in strain D14 and two more copies in strains 60 and 102, whereas no variation was observed in haploid strain W303. These enzymes are involved in the utilization of SAM as a sulfur source, and also in glutathione (GSH) biosynthesis, which is the main antioxidant molecule in yeast cells. As the oxidative stress response is very important for opportunistic yeasts to survive in human blood, we studied if these strains were capable of increasing the GSH content using SAM as a sulfur source. However, no differences were observed (results not shown). In fact, *SAM3* and *SAM4* are located in a subtelomeric cluster that has been shown to have higher CNs in other strains [31]. Therefore, we cannot rule out that this genomic alteration is not a specific feature of the opportunistic strains.

### Genetic changes in the *de novo* purine nucleotides biosynthetic pathway

We focused our study on *IMD2*, a gene showing increased CNs in the three opportunistic strains compared with laboratory strains (Fig. 2A). This gene belongs to the *IMD* gene family (*IMD1-4*), which code for Inosine monophosphate dehydrogenases (IMD), enzymes which catalyzes the rate-limiting step in the *de novo* GTP biosynthetic pathway. We confirmed the increased CNs of *IMD2* by qPCR, and showed at least two more copies of the gene in strain 102 and three more copies in strains 60 and D14 (Fig. 2C). Similar alterations were monitored for its closely related gene, *IMD3*, where increased CN was observed in strains 102 and D14 (Fig. 2A), but not in strain 60. These results were also confirmed by qPCR, showing at least one more copy of *IMD3* in strains 102 and D14 (Fig. 2C). No signal was detected for *IMD3* amplification in strain 60.

We wondered if the increased CNs observed for the *IMD* genes in the opportunistic strains could be a result of their adaptation or ability to survive in human environments and whether this could be associated with their ability to cause infections. *IMD2* is a subtelomeric gene, which has been correlated with altered CNs in strains of different origins. In fact, other genes located next to *IMD2* exhibit CNVs (Fig. 1E). In contrast, *IMD3* is localized at the right arm of chromosome XII and is not close to any ribosomal genes, transposons or any other genomic feature that are known to favor recombination or genome rearrangements. Furthermore, no other genes close to *IMD3* have been shown to have altered CNs (Fig. 1E). We studied the CNVs of the other genes implicated in the *de novo* biosynthesis of the purine nucleotides pathway (Fig. 2D) and we observed increased CN for other genes, such as *GUK1* in strain 60 and *RNR4* in 102. No significant increased CN was observed in the W303 strain and no significant decrease in any gene related to the purine nucleotides pathway was found. All these data suggest that this pathway might be of special importance for the opportunistic strains to develop their specific abilities.

In order to test the role of the *de novo* biosynthesis of the purine nucleotides pathway in *S*. *cerevisiae* virulence, we wanted to construct a knock out mutant that is unable to use this pathway within the genetic background of the opportunistic strain D14. Deleting the *IMD* genes, which are redundant among them and showed increased CN, can be a complex approach (around 10–14 copies can be found, 2 of each IMD1-4 gene and extra copies depending on the strain). Instead of we selected gene *GUA1* of D14, because Gua1p catalyzes the subsequent step of the Imd enzymes, and because no increased copy numbers of *GUA1* exist in D14. In fact, and as expected, the deletion of two *GUA1* copies in the diploid strain D14 led to a *GUA1* knock out mutant (D14Δgua1). We also constructed a reconstituted *GUA1* (D14GUA1) strain as a control by transforming the D14Δgua1 mutant with an episomal plasmid containing *GUA1*.

**Fig 3:**
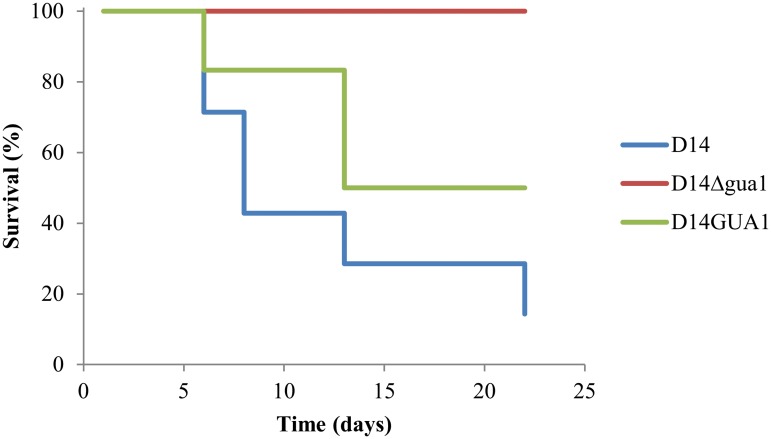
Virulence implication in *S*. *cerevisiae* opportunistic strain D14 of the *GUA1* gene. *GUA1* encodes an essential enzyme of de novo biosynthesis of purine nucleotides pathway in immunocompromised murine models. DBA/2 healthy mice (8 for each strain) were intravenous inoculated with the corresponding yeast strain and survival was followed every day. Mouse immunodeficiency was produced by intraperitoneal injections of cyclophosphamide (150 mg/kg) at day -1 and 5 after yeast infection. Kaplan-Meyer curves were represented. The *GUA1*-reconstituted strain survival was no significantly different (*p*-value<0.05) to the parental strain, but the *GUA1*-reconstituted strain and D14Δgua1 strain were significantly different (*p*-value<0.05) to the parental strain.

Next, we evaluated the virulence of these two strains and the wild-type D14 in murine infection models (Fig. 3). Since *S*. *cerevisiae* infections have been described mainly in immunodeficient patients, we used a mice model of systemic infection with cyclophosphamide-induced neutropenia, which has been previously been used in *S*. *cerevisiae* and in other pathogenic organisms such as *C*. *albicans* [16], [24], [32]. The results revealed that only 15% of mice infected with strain D14 survive until day 22, whereas D14Δgua1 was unable to cause death in the mice. The *GUA1*-reconstituted strain caused a similar pattern (no significantly different, *p*-value<0.05) of mice survival as compared to the parental strain, but was moderately reduced in virulence (50% survival of mice). On the contrary, The *GUA1*-reconstituted strain and D14Δgua1 strain were significantly different (*p*-value<0.05) to the wild type strain.

**Fig 4:**
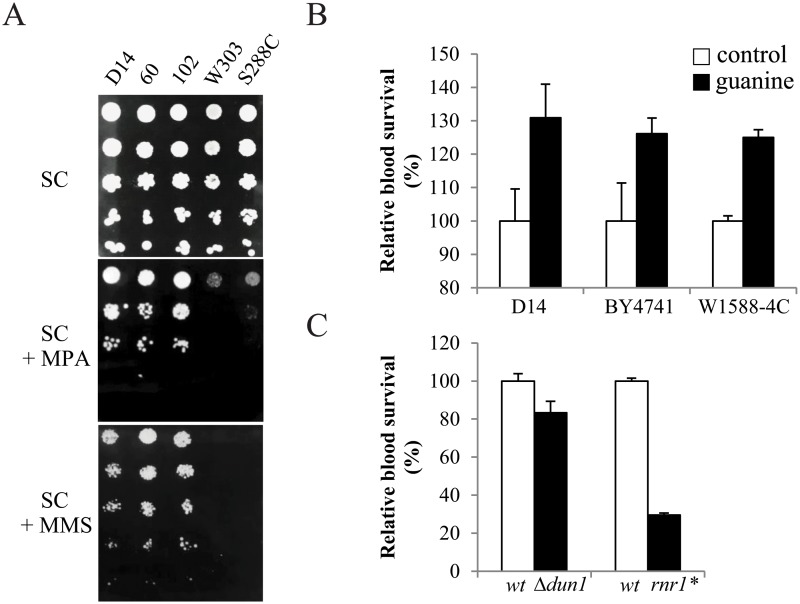
The critical role of genes related to dNTP pool increase in response to human blood phagocytes. A) Evaluation of yeast performance in response to 7 μg/ml of mycophenolic acid (MPA) or 0.004% of MMS was compared to control minimal media SC for opportunistic strains D14, 102 and 60 and for control lab strains S288C and W303. B) Evaluation of yeast survival after 90 min of *ex vivo* human blood incubation. After blood incubation, wild type lab strains BY4741 and W1588-4C and D14 opportunistic strain were plated in YPD or YPD with guanine (65 μM) and colony were counted. C) Also, dun1 and rnr1* mutants were plated in YPD after blood exposure. Triplicates average and standard deviation of normalized survival data (YPD + guanine respect YPD and mutants respect its isogenic parental wild type) is represented. In all cases (control respect guanine and mutants respect to wild type) differences were significantly different using *p*-value of 0.05.

Decreased survival in D14Δgua1 mice can be a consequence of low guanine levels in the host. However, an increased dNTP pool which could be used for DNA repair. An increased DNA repair may be required during systemic infections since oxidative damage of yeast DNA is one of the key events which occur after blood infection. To test this hypothesis, we studied yeast growth in the presence of mycophenolic acid (MPA), an inhibitor of Imd enzymes. In fact, the expression of *IMD* genes is induced by mycophenolic acid to result in resistance to the drug [23], [33], [34]. The results (Fig. 4A) reveal that the opportunistic strains are able to grow in the presence of 7 μg/ml of MPA, whereas the growth of laboratory strains S288C and W303 was strongly affected. These results phenotypically confirm the predictions made based on the CGH and qPCR data. Next, we tested the growth of the strains in the presence of the DNA damaging agent methyl methanesulfonate (MMS), which produces alkylated DNA that is poorly replicated by DNA polymerases and must be efficiently repaired. We observed that the laboratory yeast strains S288c or W303 were highly sensitive to MMS (0.004%) treatment, whereas the opportunistic yeasts strains were much less affected (Fig. 4).

In order to study the role of the *novo* purine nucleotide biosynthesis pathway when yeast cells face human phagocytes, we quantified fungal survival after *ex vivo* human blood infection when neutrophils attack yeast cells and produce reactive oxygen species. We studied yeast survival after 90 min of blood incubation by colony counting on rich media. This experimental set up was already used to show increased survival of opportunistic yeasts compared to other strains in blood infections [25]. Here we observed that addition of guanine to rich media increased survival in the opportunistic strain D14, but also in the laboratory wild-type strains BY4741 and W1588 (selected to compare with their isogenic mutants, see below) (Fig. 4B). In order to ensure that increased survival was not due to an additional and more efficient nitrogen source, we performed the same experiment adding the equivalent amount of nitrogen. However, no increased survival of yeast cells was found (data not shown). In this assay, we also analyzed the behavior of two mutants with lower dNTP levels due to mutation of genes which influence the expression of the last enzyme of the dNTP biosynthetic pathway, ribonucleotide reductase (RNR). One mutant tested was Δdun1, which lacks the *DUN1* gene. *DUN1* encodes a key activator of RNR in response to DNA damage. The Δ*dun1* mutant was previously shown to have dysfunctions in RNR activation and decreased dNTP pools after DNA damage [34], [35]. The other mutant tested was rnr1*, containing a point mutation that disrupts RNR activation in response to DNA damage [19]. The results (Fig. 4B) indicate that both mutants were less able to survive in human blood as compared to their isogenic parental wild type strains. Especially rnr1* had a strongly (more than 70%) reduced ability to survive the challenge of blood cells. These data suggest that *de novo* dNTP biosynthesis plays a role in yeast survival after DNA damage produced by neutrophils or other blood cells.

## Discussion

To elucidate the genetic determinants that enable certain strains of *S*. *cerevisiae* to cause infection, we used a comparative genetics approach. We compared the genomes of strains showing high level of virulence attributes (pseudohyphal and 42°C growth, phosphatases and proteases secretion, and the ability to infect and kill mice) independently of the isolation origin [13], [16], [18] with the gene content of non-virulent laboratory strains using microarrays. Many strains of *S*. *cerevisiae* which have been isolated in clinical settings present very low levels of virulence in mice infection models and other strains, such as strain D14, isolated from a dietetic supplement, show a relative high level of virulence in different infection models. Thus, we propose that the term “opportunistic strain” is more accurate and useful to categorize these strains rather than “clinical strains”, since not all clinical strains cause infections. We define opportunistic *S*. *cerevisiae* strains as those strains which can, in contrast to most other strains, cause infections in mice and can kill mice. These strains also survive better in human blood infection models than other strains, which may enable these strains to disseminate across the body and to reach organs with adequate propagation conditions under certain circumstances [25]. Thus, increased blood survival can be a key feature to distinguish these opportunistic yeasts strains from others. Previous studies by our group have demonstrated that opportunistic strains show a specific transcription pattern after human blood infection, which indicate a specific oxidative stress response and DNA damage repair [25]. DNA damage is apparently produced by reactive oxygen species generated mainly by neutrophils and a proper DNA repair may be a specific characteristic of opportunistic strains. In this study, we aimed at further characterizing these strains by using a genomic approach.

A common mechanism described for human microbial pathogens is to increase dNTP pools in order to repair DNA damage caused by the oxidative burst of phagocytes. For example, the importance of a *de novo* nucleotide biosynthetic pathway for phagocyte survival has been demonstrated for bacterial pathogens such as *Salmonella enterica* [36], *Bacteroides fragilis* [37], *Pseudomonas aeruginosa* [38], *Staphylococcus aureus* [39], *Streptococcus pyogenes* [40], *Bacillus anthracis* and *Escherichia coli* [41]. The relevance of *de novo* nucleotide biosynthetic pathways has also been implicated for fungal pathogens like *Cryptococcus neoformans* [42] or *Candida albicans* [43], [44] and nucleotide biosynthetic pathways have been discussed as a target for antifungal compounds [45]. The results obtained in our study highlight a specific genomic fingerprint of opportunistic strains that increase the gene copy number of genes involved in the *de novo* biosynthesis of purine nucleotides. We conclude that *S*. *cerevisiae* opportunistic strains, like other human pathogens, have the enhanced ability to produce dNTPs, the substrates that are used by DNA repair machineries. This is supported by the observation that mutantsΔdun1 and rnr*, which showed lower dNTP pools and defects in RNR activation after DNA damage [19], [34], [35], display poorer survival in human blood as compared to wild type strains. Other genes involved in DNA repair are also highlighted by our CGH study. One interesting example is *IMP2*, a transcription factor gene, which is activated in response to DNA damage produced by oxidative stress and which regulates the expression of DNA repair genes. Thus, an enhanced dNTP production pathway combined with efficient DNA repair machinery may be a key feature to define opportunistic *S*. *cerevisiae* yeasts. Our study opens up the possibility the *de novo* purine biosynthetic pathway is a possible alternative target to treat *S*. *cerevisiae* infections, as previously proposed for other fungal pathogens such as *C*. *albicans* [43–45].

In general terms, increased dNTP biosynthesis to supply DNA repair seems to be a general strategy used by some pathogens to survive human macrophages oxidative burst. However, those genes that have been shown to be associated with virulence differ among the different organisms. The role of Imd activity, which is the rate-limiting step in the *de novo* purine biosynthetic pathway, has been stressed in the fungal pathogen *C*. *neoformans* [42]. In *C*. *albicans*, other genes, or genes associated with the same pathway, for example *ADE2*, *ADE7* or *GUA1*, have also been implicated in virulence [43], [44]. In contrast, studies dealing with bacterial pathogens have proposed that RNR activity is most important, since it is the key rate-limiting step in deoxyribonucleotides formation [37–39]. Here we propose that both *IMD* genes and RNR are important for opportunistic yeasts to cause infections. The fact that opportunistic yeasts have increased *IMD* gene copy numbers suggests that the level of such activity is crucial, although we also observed that diminished RNR activity dramatically affects survival in blood infections. Therefore, both activities, Imd and RNR, influence the ability of opportunistic *S*. *cerevisiae* strains to overcome an oxidative burst attack by phagocytes and to survive at least transiently in blood. This property may enable these strains to reach other organs and to proliferate.

New knowledge on opportunistic yeast is essential to understand the intrinsic differences between these strains and the standard strains used in the food industry considered safe. This will help in the selection of strains that are suitable to produce foods. Some criteria has been previously suggested to avoid opportunistic yeast in foods as growth above 37°C [46], [47] or increased oxidative stress resistance [25]. Here we propose new criteria to discard opportunistic strains for the food industry: increased resistance to DNA damage agents or increased resistance to *de novo* dNTP biosynthesis inhibitors. A combination of all this criteria will be useful to select safer strains for the production of human foods.
